# Particular vulnerability of patients with borderline personality disorder during the COVID-19 pandemic – a retrospective chart review

**DOI:** 10.1186/s12888-024-06366-y

**Published:** 2024-12-27

**Authors:** Yann David Kippe, Stefan Gutwinski, Maia Adam, Anna Finck, Meryam Schouler-Ocak, Thomas Goldschmidt

**Affiliations:** Psychiatrische Universitätsklinik der Charité im St. Hedwig Krankenhaus, Große Hamburger Str. 5-11, Berlin, 10115 Germany

**Keywords:** Borderline personality disorder, COVID-19 pandemic, Suicide attempt, Psychiatric emergency department, Social distancing

## Abstract

**Background:**

Mental health consequences of the COVID-19 pandemic have been a major research focus since its beginning. A specific vulnerability of patients with borderline personality disorder (BPD) following social distancing measures has been reported, however there is a lack of adequately sized studies that provide evidence of this vulnerability. Suicide attempts may reflect mental health effects of the COVID-19 pandemic in psychiatric settings.

**Methods:**

Retrospective follow-up analysis of clinical documentation in a psychiatric emergency department (pED) of a major academic psychiatric hospital in Berlin, Germany. Observation periods include the first- (3/2/2020–5/24/2020) and second-wave (9/15/2020–3/1/2021) of the COVID-19 pandemic in Germany and respective periods one year earlier as control-periods. Poisson-regression was used for statistical modelling of individual counts of pED presentations after a suicide attempt.

**Results:**

*N* = 4110 patients attended the pED during the four observation periods. BPD patients were associated with elevated risk of pED presentation after a suicide attempt during COVID-periods (RR = 3.4; *p* = .014). Schizophrenia and psychotic disorders showed lower risk of pED presentation after a suicide attempt during COVID-periods (RR = 0.4; *p* = .048). Other diagnostic groups did not show significant interaction effects with COVID-periods. The first-wave was a risk factor for pED presentation after a suicide attempt affecting the sample across all diagnostic groups (RR = 3.1; *p* = .006).

**Conclusions:**

BPD patients seem to be particularly vulnerable during the COVID-19 pandemic showing increased rates of suicide attempts during both COVID-periods. This should be addressed in future health crises by ensuring availability of psychosocial help. There is a need for further research regarding BPD patients in public health crisis situations.

**Supplementary Information:**

The online version contains supplementary material available at 10.1186/s12888-024-06366-y.

## Introduction

The COVID-19 pandemic and its mental health sequelae in the general population have been a major research focus since the declaration of a public health emergency in early 2020 [1]. While there has been a negative impact of the COVID-19 pandemic on mental health in the general public, it seems that this has been limited to the first phase of the pandemic and that mental health symptoms were back on pre-pandemic levels in mid-2020 [2]. Among the most severe negative outcomes in terms of mental health are suicidal behaviours (SB) and death by suicide. Various psychosocial issues originating from social distancing measures and lockdowns may have increased risk factors for SB such as loneliness or despair. Their impact, probably, was particularly severe in populations that were vulnerable already in prepandemic times [3]. Regarding SB, although with some heterogeneity [4], the COVID-19 pandemic seems to not have led to a rise in suicidal ideation (SI) or SB in the general population [5]. In a large-scale analysis of suicide mortality data, no rise in deaths by suicides could be detected [6]. There are reports that some subgroups may be disproportionately affected by the COVID-19 pandemic [2, 7]. Among those are patients with Borderline Personality Disorder (BPD), who may be especially vulnerable to social isolation due to their specific psychopathology [8], which involves difficulties regarding interpersonal relationships, extreme affective lability and impulsivity [9]. COVID infection containment strategies, moderated through social isolation, may have led to an increase in maladaptive coping strategies in patients with BPD, such as increased substance use, self-injury or SB [8, 10].

Although this particular vulnerability has been described theoretically [8], the literature on this subject is very scarce up to date: a case series with *N* = 50 patients in Spain reported no worsening of BPD symptoms [11]. Nonetheless, living alone was shown to be the most important predictor of worse clinical outcome [11]. Another study investigated the effect of the COVID-19 pandemic using semi-structured clinical interviews *N* = 16 patients with emotionally unstable personality disorder and reported an increase in SI and substance use [10]. A symptom diary analysis of *N* = 7 BPD patients comparing 8 weeks prior to social distancing measures to 8 weeks with implemented contact ban measures showed less overall tension but increased general distress [12]. All of these studies had small case numbers and – except the last – share a lack of comparison with a pre-pandemic control group.

The objective of this follow-up analysis is to provide an accurate picture of how the COVID-19 pandemic influenced presentations to a psychiatric emergency department (pED) after a suicide attempt (SA) in a large sample of patients from an academic psychiatric hospital in Berlin with a special focus on patients with BPD.

Analysing the occurrence of SA in a pED may help to gain valuable insight into the mental health of psychiatric patients in general and BPD patients in specific [13]. In a previous publication by the authors of this study, the first wave of the pandemic in Berlin was estimated to lead to a 9.9-fold increase of the individual risk of presenting to the pED after a SA, while psychiatric diagnoses during the first wave did not add to the risk. During the second wave of the pandemic, only patients with BPD displayed an elevated risk for a presentation at a pED after SA [7]. The above-mentioned indications of particular vulnerability of BPD patients and the option of possible errors in the estimation of effect sizes by executing the analysis separately for both waves, led us to perform this follow-up analysis. It addresses the limitations of the previously used regression models by combining both waves in order to retrieve more robust effect size estimations.

## Methods

The dataset of this study has been acquired by retrospective data extraction from clinical records of all patients presenting to the psychiatric emergency department (pED) of a large academic psychiatric hospital in Berlin, Germany during the first (3/2/2020 – 5/24/2020 “first-wave”) and the second wave (9/15/2020 – 3/1/2021 “second-wave”) of the COVID-19 pandemic; the same periods one year earlier were used as control-periods [7]. Characteristics of in- and excluded pED presentations can be found in supplementary material S1. To reduce possible interrater bias and to ensure a uniform evaluation of cases, training in data extraction from clinical documentation files and close coordination between the investigators took place. Ambiguously documented cases were evaluated in case discussions in consensus of all investigators. For analytic purposes, we clustered diagnoses of the International Statistical Classification of Diseases and Related Health Problems, 10th revision (ICD-10), into diagnostic groups. A detailed description of the diagnostic groups can be found in supplementary material S2. For more information on the dataset see a prior publication by the authors [7]. The study was approved by the local ethics committee (Ethikkommission der Charité – Universitätsmedizin Berlin; number of approval: EA 110/20) and has been performed in accordance with ethical standards of the declaration of Helsinki.

### Statistical analysis

The statistical model used in this study is a Poisson-regression with the outcome variable being the individual number of suicide attempts (SA) per patient within one observation period. To retrieve the outcome variable, a transformation of the data from long- to wide-format had to be performed (see [7]). To test Poisson-distribution of the outcome variable, a Kolmogorov–Smirnov test was performed. In order to test the hypothesis of longitudinal diagnosis-specific effects of the COVID-19 pandemic, we joined data of all four observation periods (first-wave, second-wave and corresponding control-periods) for the statistical model, introducing an offset variable accounting for differences in period lengths. Effect sizes are reported as Rate Ratios (RR) with 95% confidence intervals (95%CI). The overall significance level was set to *p* < 0.05. The investigated effects were modelled using interaction effects with COVID-19 pandemic:

The general impact of the pandemic in either of the observed waves is being estimated by interaction of first-wave or second-wave by COVID-period. This accounts for all differences between the two waves, which were characterized by different regimes of infection mitigation measures, different infection incidence numbers and seasonal differences.

To examine effects of the COVID-19 pandemic specific to diagnostic subgroups, interaction effects between diagnostic groups and COVID-periods were used in the statistical model. We hypothesized that the specific effect in the BPD group originates from the specific psychopathology, thus the effect was hypothesized to be present longitudinally during both observed COVID-periods. Accordingly, we modelled the interaction effect between diagnostic subgroups and the combined COVID-periods. The model estimates effect sizes of the effect of the COVID-19 pandemic separately for each diagnostic group. Diagnostic groups which were included in these interaction effects as of their association with suicidality are: Borderline personality disorder (BPD) [14–16], substance use disorders [14], schizophrenia and psychotic disorders [14, 17], bipolar and manic disorders [14, 18, 19] and unipolar depressive disorders [14, 18, 19].

In the previous statistical analysis [7], there was a possible overlay between short- and long-term effects leading to errors in effect size estimates. In contrast, the approach of this follow-up analysis is enabling the Poisson-regression model to differentiate between short- and longterm effects, allowing for more robust effect size estimations.

To account for unevenly distributed risk of SA between different diagnostic groups, we time-independently controlled for diagnostic groups. We included time-independent effects of age and gender, but in contrast to the previous publication we decided to not control for other sociodemographic factors, since these did not show a significant influence on the number of presentations after SA [7].

A negative-binomial regression was performed as a sensitivity analysis (supplementary material S3, S4 and S5). Furthermore, a sensitivity analysis excluding patients with multiple SA within one period was performed (supplementary material S6). All statistical analyses were performed using the SPSS Statistics package, version 27.0, IBM Corporation (2020).

## Results

In this follow-up analysis, the same amount of *N* = 5634 pED presentations during the four observation periods were included as in the previous analysis [7]. The total number of patients presenting to the pED was *N* = 4110, *N* = 168 patients presented to the pED after a SA, of which *N* = 4 patients presented to the pED after a SA twice within one observation period, two being the maximum number of pED presentations after SA in individual patients (Table 1). The results of the sensitivity analysis excluding patients with multiple SA in one period did not show major differences to the main analysis of this study (supplementary material S5). Table 1 displays demographic and clinical characteristics of all patients in our sample. For a description of patient characteristics within each observation period separately, please refer to the previous publication by the authors [7].

**Table 1:** Characteristics of patients presenting to the pED

	**control periods**	**COVID-19 periods**	**difference**	***p*-value**	
*N total number of patients*	2189	1921	−12,2%		
*Median age*	39 years	39 years	+ −0 years	.916	Z = -.105
*Female gender (%)*	905 (41.3%)	783 (40.8%)	−13.5%	.715	
*mean number of pED presentations per patient (SD)*	1.31 (.871)	1.44 (1.262)	+ 9.9%	** < .001**	
*n number of patients with pED presentation after SA (%)*	78 (3.6%)	90 (4.7%)	+ 15.4%	.070	
*n number of patients with 2 pED presentations after SA (%)*	0 (0.0%)	4 (0.2%)			
**Diagnostic categories**					
* Borderline personality disorder (%)*	172 (7.9%)	133 (6.9%)	−22.7%	.254	
* Other personality disorders (%)*	95 (4.3%)	83 (4.3%)	−12.6%	.976	
* Personality disorders combined (%)*	267 (12.2%)	216 (11.2%)	−19.1%	.344	
* Organic mental disorders (%)*	127 (5.8%)	114 (5.9%)	−10.2%	.857	
* Substance use disorders (%)*	1056 (48.2%)	900 (46.9%)	−14.7%	.373	
* Schizophrenia and psychotic disorders (%)*	578 (26.4%)	592 (30.8%)	+ 2.4%	**.002**	
* Bipolar and manic disorders (%)*	116 (5.3%)	106 (5.5%)	−8.6%	.757	
* Depressive disorders (%)*	453 (20.7%)	338 (17.6%)	−25.4%	**.012**	
* Neurotic, somatoform and stress related disorders (%)*	512 (23.4%)	416 (21.7%)	−18.8%	.185	

Comparison of demographic and clinical characteristics of patients presenting to the psychiatric emergency department in the COVID-19 periods and control periods. Difference is the change of case numbers in the COVID-19 periods compared to the control periods in percentages. *P*-values are derived from chi^2^-tests, except for "median age", which were tested using the Mann–Whitney-U-test and for "mean number of pED presentations per patient", which were tested using a student's t-test. *Abbreviations used*: *pED* psychiatric emergency department, *N*, *n* patient numbers, *SA* suicide attempt, *COVID-19* coronavirus disease 2019, *SD* standard deviation

The Poisson regression analysis of the interaction effect of BPD and COVID-periods (first-wave and second-wave combined = longitudinal effect) estimated an elevated risk of RR = 3.406 (95%CI: 1.283–9.043; *p* = 0.014, Table 2) in the BPD patient group. The interaction effect of schizophrenia and psychotic disorders and COVID-period was associated with a lower risk of presenting to the pED after SA (RR = 0.432; 95%CI: 0.188–0.994; *p* = 0.048, Table 2). COVID-dependent interaction effects in other diagnostic groups did not affect the individual number of pED presentations after SA. The first-wave of the COVID-19 pandemic was an independent risk factor for a pED presentation after SA (RR = 3.129; 95%CI: 1.391–7.038; *p* = 0.006, Table 2), whereas the second-wave alone was not associated with elevated risk for a presentation after SA.

**Table 2:** Poisson model estimating effects of COVID-19 periods on suicide attempts including interaction effects

	**RateRatio**	**95% CI lower**	**95%CI upper**	***p*-value**
**Time-dependent**				
* first-wave by COVID-19*	3.129	1.391	7.038	**.006**
* second-wave by COVID-19*	1.499	0.695	3.232	.302
* Borderline personality disorder by COVID-19*	3.406	1.283	9.043	**.014**
* Substance use disorders by COVID-19*	0.974	0.525	1.807	.934
* Schizophrenia and psychotic disorders by COVID-19*	0.432	0.188	0.994	**.048**
* Bipolar and manic disorders by COVID-19*	0.295	0.026	3.378	.327
* Depressive disorders by COVID-19*	0.502	0.248	1.018	.056
**Time-independent**				
* Borderline personality disorder*	1.178	0.497	2.788	.710
* Other personality disorders*	1.815	1.021	3.225	**.042**
* Organic mental disorders*	1.041	0.484	2.241	.918
* Substance use disorders*	1.264	0.787	2.029	.333
* Schizophrenia and psychotic disorders*	1.107	0.608	2.014	.740
* Bipolar and manic disorders*	0.659	0.157	2.755	.567
* Depressive disorders*	2.719	1.636	4.518	** < .001**
* Neurotic, somatoform and stress related disorders*	0.956	0.649	1.408	.820
* Age*	1.001	0.990	1.011	.905
* gender*	1.268	0.910	1.767	.160

Results from the Poisson regression models with the number of pED presentations after a suicide attempt per patient and observation period as outcome variable. Rate ratios greater than 1 indicate that a factor is increasing the number of presentations per patient after a suicide attempt. Rate ratios below 1 indicate that a factor is decreasing the number of presentations per patient after a suicide attempt. *Abbreviations used:* *95% CI* 95% confidence interval, *COVID-19* coronavirus disease 2019

Regarding time-independent effects, the model estimated patients with depressive disorders to be at elevated risk for a pED presentation after SA (RR = 2.719; 95%CI: 1.636–4.518; *p* < 0.001, Table 2). Furthermore, other personality disorders were associated with elevated risk for a pED presentation after SA (RR = 1.815; 95%CI: 1.021–3.225; *p* = 0.042, Table 2). Other time-independent factors that we controlled for did not significantly influence the individual number of pED presentations after SA (Table 2).

## Discussion

Our findings, based on the first two waves of the COVID-19 pandemic in Berlin, show that patients with BPD were associated with a 3.4-times increased risk of a pED presentation after SA during the COVID-19 pandemic. A general effect of the COVID-19 pandemic was seen for the first-wave, being associated with a 3.1-times increased risk for a pED presentation after SA across all diagnostic groups. Patients with schizophrenia and psychotic disorders were associated with a decreased risk for a pED presentation after SA during the observed COVID-periods.

### Borderline personality disorder

The specific effect for patients with BPD estimated a 3.4-times increased risk for a pED presentation after SA during the observed COVID-periods, showing the highest elevation of all examined diagnosis groups. In previous analyses of the same sample, which modelled the two observed COVID-periods with their control periods separately, this BPD specific effect was only apparent during the second-wave of the COVID-19 pandemic, with a rate ratio (= relative risk) of 7.1 [7]. It is likely that due to separate modelling of the two infection waves, the BPD specific effect during the first-wave was included in the general effect (diagnosis independent) of the first-wave, since the model could not differentiate between short-term (occurring only in one COVID-period) and longitudinal effects (occurring in both COVID-periods) effects. Accordingly, the risk ratio for the first-wave is now being estimated at 3.1, in contrast to the first-wave being attributed with a risk ratio of 9.9 in the previous publication [7].

The finding of a longitudinally increased risk for SA in patients with BPD is a new and interesting result, highlighting the vulnerability of this patient group, which has until date not been shown in a large clinical sample. In their narrative review, Preti et al. provide a good synopsis of the vulnerability mechanisms: social distancing measures, which at its core share the reduction of physical contacts, conflict with a “strong need for emotional and physical proximity with others” [8] and may aggravate fears of abandonment and rejection, which are characteristic for BPD [8, 10]. This may lead to an increase in dysfunctional coping mechanisms such as substance abuse, non-suicidal self-injury or SB [8], which could also be shown in a small series (*n* = 16) of semi-structured interviews in BPD patients [10]. Another aggravating factor may have been introduced through less social support and its subsequently decreased moderating effect on impulsivity [7]. A concept, that is worth discussing, is the feeling of emptiness, which is a “complex, negative emotional state” [20], characterized by “aloneness or social disconnectedness” [20] and “a deep sense of personal unfulfillment or lack of purpose” [20]. Chronic emptiness is prevalent in many BPD patients and is a diagnostic criterion for BPD in the Diagnostic and Statistical Manual of Mental Disorders (DSM-V) [9]. The prolonged time, in which social distancing measures were in effect, but also the closing of many leisure activities and the medial presence of the pandemic may have contributed to aggravating emptiness in BPD patients. This may give an explanation to our results indicating an elevated risk for SA in this patient group, since chronic emptiness is the BPD feature most strongly associated with psychosocial morbidity, acute suicidality and a history of SA [21–24]. A lack of meaning in life as well as demoralization might, as Constanza et al. postulate, mediate between the presence of risk factors for suicide according to the interpersonal theory of suicide and the occurrence of SI and SA [25]. Social distancing measures may have enhanced doubts about the meaning in life as well as a demoralization, which may have been experienced more intensely by BPD patients, further explaining their specific vulnerability. However, more studies are needed to examine this mediation role.

Studies on the fate of patients with BPD during the pandemic include a case series [11] and a diary analysis [12], which have been described in the introduction section of this paper. Patten et al. analysed hospital discharge data from the province of Alberta, Canada, finding an increase in hospital admissions of patients with personality disorders during the COVID-19 pandemic [26]. This indicates an increased demand for support during the pandemic and fits into the theory of a vulnerability of BPD patients, which represented 77% of patients in the personality disorders group [26]. A survey study conducted in Norway found no change in the rate of SA in their sample of *N *= 133 patients with personality disorders, however the authors reported increased levels of anxiety, depression, aggression, substance use and social isolation [27]. These results stem from retrospective self-reports of patients who have received treatment in a network of personality disorder treatment units before the start of the pandemic and may thus represent a spectrum of patients with less severe personality disorder symptoms when compared to the acute ward setting of our study, possibly explaining the different results regarding SA outcomes. Moreover, the low response rate (12%) [27] may have resulted in a selection bias towards more functional patients. The other reported outcomes indicate difficulties of patients with personality disorders in adapting to the pandemic situation, however there was no comparison to other diagnostic groups, which limits the possibility of to draw conclusions regarding a specific vulnerability of BPD patients.

To our knowledge, there is only one study with a similar design to the current one: a study in Frankfurt, Germany examined the occurrence of SA in all psychiatric hospitals of the city and reported a decrease of SA by −31% from March to December 2020 compared to the same period in 2019 [28]. The proportion of patients living alone when committing a suicide attempt increased, however no differences regarding diagnostic groups were reported [28]. This may be due to local differences in the context of the studies such as different size of the involved cities and the composition of the study sample regarding diagnostic categories. Another factor to consider are differences in the specific study design, which prospectively excluded “self-harm for emotional tension relief without suicidal ideation e.g., in the context of borderline personality disorder” [28]. While this discrimination may be possible in a prospective design, it was not possible to assess the suicidal intent in the retrospective document analysis in the chart data of the current study, which is one of its main limitations to consider. Due to the more restrictive inclusion criteria, it seems plausible that Reif-Leonhard et al. may have classified some cases of severe self-injury as non-suicidal, that would have – due to the retrospective nature of this study – been classified as SA in the current study. In a study of Aguglia et al., various measures were used to retrospectively assess SA severity/lethality [29]. In modification of their approach, we assessed in both the total sample as well as in the BPD subgroup, whether there was a difference between observation periods in the percentage of SA admissions requiring intensive care treatment. In neither group a difference was found (see supplementary materials S7). It is difficult to draw a conclusion from this however, since the case numbers of intensive care treatments were low. One reason may be that not all external intensive care treatments were recorded. Future studies of similar design to ours should consider implementing ways to differentiate between low and high lethality SA following Aguglia et al. [29]. However, our data may be valuable despite this missing differentiation, as both – SA and serious self-injury or parasuicidality – can be seen as a clinical endpoint of a deterioration regarding the BPD psychopathology, highlighting the specific vulnerability of BPD patients during the pandemic. It is noteworthy, that the rates of SA in BPD patients during the control periods are rather low (3.5%, cf Table 3), when compared to those in the study of Reif-Leonhard et al. (9.9% SA in the F6x diagnostic group) and in another study from France (37% SA in BPD group [30]). There are no convincing reasons to expect the year 2019 to be in any kind special for the group of BPD patients in Berlin. A small interrater bias, however, cannot be completely ruled out, although there were measures taken in order to reduce interrater bias (cf methods section). Furthermore, important site differences in suicidality in BPD have been reported earlier: Collins et al. report in their meta-analysis a rate range of 4 to 66% regarding the proportion of BPD patients of all SA in pED [31].

**Table 3:** Patients with at least one pED presentation after SA across diagnostic categories per period

	**control periods**	**COVID-periods**	***p*-value**	
**Diagnostic categories**				
* Borderline personality disorder (%)*	6 (3.5%)	20 (15.0%)	** < .001**	
* Other personality disorders (%)*	6 (6.3%)	7 (8.4%)	.588	
* Organic mental disorders (%)*	4 (3.1%)	5 (4.4%)	.739	Fisher
* Substance use disorders (%)*	40 (3.8%)	49 (5.4%)	.080	
* Schizophrenia and psychotic disorders (%)*	17 (2.9%)	13 (2.2%)	.420	
* Bipolar and manic disorders (%)*	2 (1.7%)	1 (0.9%)	1.000	Fisher
* Depressive disorders (%)*	31 (6.8%)	22 (6.5%)	.852	
* Neurotic, somatoform and stress related disorders (%)*	18 (3.5%)	20 (4.8%)	.323	

Comparison of number of patients presenting to the psychiatric emergency department after a suicide attempt within each diagnostic category in the control periods and COVID-periods. *P*-values are derived from chi^2^-tests, except where otherwise indicated. "Fisher" refers to the Fisher-exact-test and has been used when expected case numbers were < 5, thus not allowing chi^2^-tests. *Abbreviations used:* *pED* psychiatric emergency department, *SA* suicide attempt

With a total of *n* = 305 BPD patients, the current study is the largest clinical sample to date in which the vulnerability of BPD patients during the COVID-19 pandemic is apparent, providing valuable evidence that support the theoretical mechanisms described in the literature [8, 32]. However, more evidence needs to be accumulated to further confirm the vulnerability of patients with BPD in the context of social distancing measures. Specifically, there is a need for studies that include multiple control periods spanning several years to increase the robustness of results. In addition, the relationship between social isolation and chronic emptiness should be examined by future research.

### General effect of the pandemic

Our results indicate a 3.1-times increased risk for a pED presentation after SA during the first-wave of the COVID-19 pandemic, whereas the second-wave did not show an elevated risk. As expected, the effect size for the first-wave is smaller than in the previous analysis of the same data using the approach of separate modelling the two infection waves, which indicated a 9.9-times increased general risk for a pED presentation after SA during the first-wave [7]. As elaborated above, this is likely due to the possibility for the model to differentiate between short-term (effects apparent only in one wave) and long-term (effects that are apparent in both COVID-19 periods) effects. Since the general effect is influencing the sample across all diagnostic groups, it seems likely that an explanation may be found in external circumstances rather than in individual psychopathology.

In the light of the current body of evidence, which indicates that no change in the numbers of completed suicides [6, 28, 33–35] and – although the picture is not as clear – no general increase in the number of SA took place during the pandemic [5], the increase in pED presentations after SA may represent a shift in help seeking behaviour of patients. During the first-wave, many outpatient services closed as a reaction to the pandemic (Table 4), therefore patients experiencing suicidal ideation may have not had access to low-threshold psychological help provided by outpatient services, which would allow for a quick intervention. Potentially, these patients presented subsequently to the pED after a SA. During the second-wave, many outpatient services resumed to their regular operation (Table 4), which is also reflected by our results, indicating that there was no increased general risk of a pED presentation after SA associated with the second-wave. Other studies however indicate, that increases in SB during lockdown periods persist in post-lockdown periods [36], which have not been included in this study. Especially persons from migrant communities seem to be prone a prolonged negative mental health outcome post-lockdown as a result of the pandemic and subsequent social distancing measures [36]. Another factor, which needs to be considered when interpreting the result of a generally elevated risk for a pED presentation after SA during the first-wave, is the relatively low rate of such pED presentations in the control period of the first-wave (2.1% as compared to 4.1% during the control period of the second wave, data not shown, cf. Table 1 of the previous publication [7]). This may be due to circumstances specific to the control period for the first-wave and may lead to an overestimation of the general effect of the pandemic.

**Table 4:**
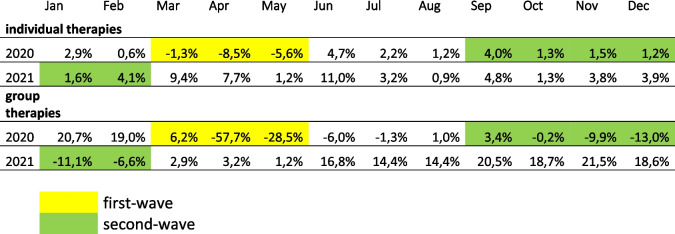
Relative number of psychotherapy provided to patients in 2020 and 2021 compared to 2019 in Germany

Relative numbers of conducted psychotherapy sessions (individual and group therapy) per month in 2020 and 2021 compared to 2019 in Germany. Months corresponding to the first-wave observation period are highlighted yellow, months corresponding to the second-wave observation period are highlighted green. *Abbreviations used:* *Jan* January, *Feb* February, *Mar* March, *Apr* April, *Jun* June, *Jul* July, *Aug* August, *Sep* September, *Oct* October, *Nov* November, *Dec* December. Data from "Zentralinstitut für die kassenärztliche Versorgung in der Bundesrepublik Deutschland", published online: Mangiapane et al., 2022 [37]

### Schizophrenia and psychotic disorders

Our model estimated patients with schizophrenia and psychotic disorders to have a lower risk (RR = 0.432, *p* = 0.048) of presenting to the pED after SA during the pandemic compared to the control periods. In the previous publication, the lower risk was only seen during the second and not during the first COVID-19 period. Overall, the proportion of patients in this diagnostic group in our sample increased during the observed COVID-periods [7, 38], indicating that these patients were presenting to the pED for other reasons than suicidality. One explanation may lie in the closing of many psychosocial care institutions and other offerings to provide support in daily life situations [39]. The role of patients with schizophrenia and psychotic disorders during the pandemic is not well understood: while some report improvements in subjective wellbeing in this cohort [40], others report a relative increase in pED consultations by patients with schizophrenia or acute psychosis [41, 42]. The retrospective approach of this study limits the possibility to draw conclusions from this result. To elaborate on the specific situation of patients with schizophrenia and psychotic disorders during the pandemic should be a focus of future research, ideally with a prospective design.

## Strengths and limitations

A major strength of this study is the large sample size compared to other studies focusing on BPD patients during the pandemic. Moreover, the combination of different COVID-periods into one statistical model allows the discrimination between short-term (occurring only in one COVID-period) and longitudinal effects (occurring in both COVID-periods) of the COVID-19 pandemic. Another strength is the patient-per-period data format used in this study, allowing risk factor estimates on an individual level.

There are some limitations to be considered: this retrospective study was conducted in a single psychiatric hospital, thus some of the observed effects might be specific to local context and extrapolation of results should be conducted carefully. Due to the retrospective design of this study, data quality relies on the routine data documentation by the psychiatric clinicians, which may be a source of bias. Discrimination between parasuicidal behaviour and SA may not have been as accurate as it may be possible in a prospective study design. The control periods of this study are limited to one year earlier, imposing the risk that our results may be biased by specific circumstances in the control periods. Data extraction from clinical documentation files was executed by MA, AF and YK under the supervision of TG. To address this circumstance, measures were implemented to reduce the risk of interrater bias. The special role of BPD patients during the COVID-19 pandemic should be further investigated with longer control periods, including post-pandemic data, to provide further evidence of the vulnerability in this patient group.

## Conclusion

In this follow-up study, BPD patients showed elevated risk for a pED presentation after SA during the observed COVID-periods. Our results suggest a specific vulnerability of this patient group that existed throughout a prolonged time during the pandemic. We observed a generally increased risk for presenting to the pED after SA during the first wave compared to one year earlier, which may represent a consequence of decreased accessibility and availability of outpatient mental healthcare services during the initial phase of the pandemic. A clinical implication to draw from our research is a stronger prioritization of accessibility to mental healthcare and psychosocial services in situations of crisis such as the COVID-19 pandemic. This may help to prevent SA in groups, that are already at risk due to mental illness. Social distancing measures and lockdowns should be avoided if possible and if they are really needed, their potential consequences including psychosocial deprivation should be taken into account and be monitored. This should be done also by clinicians focusing on particularly vulnerable groups. This study suggests that patients with BPD are particularly vulnerable for negative psychosocial effects of social distancing measures and lockdowns. There is a need for further research investigating the vulnerability of BPD patients during major public health crises, particularly the psychopathological mechanisms associated should be elucidated in further detail.

## Supplementary Information

Supplementary Material 1.

## Data Availability

The datasets used and analysed during the current study are available from the corresponding author on reasonable request.

## Abbreviations

95%CI: 95% confidence interval
BPD: Borderline personality disorder
COVID-19: Coronavirus disease 2019
DSM-V: Diagnostic and Statistical Manual of Mental Disorders
ICD-10: International Statistical Classification of Diseases and Related Health Problems, 10th revision
pED: psychiatric emergency department
RR: Rate Ratio
SA: Suicide attempt
SB: Suicidal behaviour
SI: Suicidal ideation
