# Supplementary material for: Particular vulnerability of patients with borderline personality disorder during the COVID-19 pandemic – a retrospective chart review
Source: BMC Psychiatry. 2024 Dec 27;24:950. doi: 10.1186/s12888-024-06366-y (PMC11673360; doi:10.1186/s12888-024-06366-y)
Supplement: Supplementary file 1 — Supplementary Material 1. [file 12888_2024_6366_MOESM1_ESM.docx]

**Supplementary material to “Particular vulnerability of patients with borderline personality disorder during the COVID-19 pandemic: a retrospective chart review?”**

**Yann David Kippe** ^1^**, Stefan Gutwinski** ^1^**, Maia Adam** ^1^**, Anna Finck** ^1^**, Meryam Schouler-Ocak** ^1^**, Thomas Goldschmidt** ^1^

^1 Psychiatrische Universitätsklinik der Charité im St. Hedwig Krankenhaus, Berlin, Germany^

**Corresponding Author:**  Yann David Kippe

E-Mail: yann-david.kippe@charite.de

**Contents:**

**S1 Overview of all excluded cases**

**S2 Composition of diagnostic categories**

**S3 Methods addendum**

**S4 Goodness of fit parameters of regression models**

**S5 Sensitivity analysis: Negative-binomial regression model**

**S6 Sensitivity analysis: Poisson regression model excluding patients with multiple suicide attempts**

**S7 SA admissions requiring intensive care treatment**

**S1: Overview of all excluded cases**

Cases were excluded if they concerned a day therapy unit (which were shut down during the beginning of the pandemic), if they left without being seen by a psychiatrist, if no documentation was available on suicidality or if no psychiatric F-diagnosis according to the International Statistical Classification of Diseases and Related Health Problems, 10th revision (ICD-10) was documented. For transformation of our data from event- to patient-based format, three additional cases had to be excluded because of unknown identity. Considering high-frequent attenders and the possible bias this group would impose, we merged pED presentations with subsequent hospital admissions if they were separated by less than 3 days. If cases were separated by 4-7 days, they were only merged if discharge was due to somatic complications or against documented advise of medical staff. An overview of all excluded and merged cases can be found below.

**N = 6948** **pED presentations during the 4 observation periods**

**N = 6904** pED presentations after excluding duplicate clinical records

**N = 6366** pED presentations after filtering for exclusion criteria

**N = 6043** pED presentations included after merging cases

**n = 44** pED presentations with duplicate clinical records

**n = 84** somatic presentations

**n = 31** consultations in preparation of a planned hospital admission

**n = 180** day therapy cases (patients receive treatment in hospital but sleep in their own home)

**n = 3** consultations as follow-up after hospital discharge

**n = 3** patient <18 years

**n = 3** pED presentations without clinical documentation

**n = 11** pED presentations with non-medical issues (e.g.: patients in need of shelter)

**n = 223** pED presentations with unclear information on suicidality

**n = 323** pED presentations merged:

**n = 222** cases with *0-3 days* interruption of hospital admission

**n = 61** cases with *4-7 days* interruption of hospital admission

**n = 40** pED presentations, in between two merged hospital admissions

**N = 5634 pED presentations** **included in analysis**

**n = 409** pED presentations left without being seen by medical/psychiatric staff

Abbreviations used: pED = psychiatric emergency department**S2: Composition of diagnostic categories according to ICD-10**

Organic mental disorders (OMD): F00 – F09

Substance use disorders (SUD): F10 – F19

Not included: F17 nicotine/tobacco related substance use disorders

F1x.5 substance induced psychotic disorder

F1x.7 late-onset substance induced psychotic disorder

Schizophrenia and psychotic disorders (SPD): F20 – F29

F1x.5 substance induced psychotic disorder

F1x.7 late-onset substance induced psychotic disorder

Bipolar and manic disorders (BMD): F30 – F31

Depressive disorders (DD): F32 – F33

Neurotic, somatoform and

stress related disorders (NSD): F40 – F48

Personality disorders (PD): F60 – F62

Borderline personality disorder (BPD): F60.30 and F60.31

**S3: Methods addendum**

The approach of this analysis differs from the previous analysis, in which the Poisson regression was performed on the two waves and their respective control periods separately [1]. In the current analysis, all four observation periods (first and second wave of COVID-19 pandemic and each respective control period) are merged into one model. To account for the different lengths of observation periods, an offset variable was introduced to separately estimate effect sizes of the general COVID-effect during each wave next to longitudinal diagnosis-specific effects of the COVID-19 pandemic.

A common problem occurring with the use of Poisson-regression modelling is overdispersion of the outcome variable leading to false estimates of effect sizes. Therefore, we performed a negative-binomial regression and compared the model fit using Akaike`s Information Criterion, in which the Poisson-regression model showed superior fit (supplementary material S4). The results of the negative-binomial regression are included in the supplementary materials (supplementary material S5).

**S4: Goodness of fit parameters of regression models**

|  | value/df | Pearson Chi² value/df | AIC |
| --- | --- | --- | --- |
| Poisson regression model (main analysis, table 2) | .251 | .966 | 1402.957 |
| Negative-binomial regression model (sensitivity analysis, supplementary table S4) | .220 | .926 | 1408.247 |
| Poisson regression model, excluding patients with multiple SA (sensitivity analysis, supplementary table S5) | .242 | .946 | 1356.281 |

Table S3 shows goodness of fit parameters for the regression models used in this study. Abbreviations used: AIC = Akaike’s Information Criterion; df = degrees of freedom; SA = suicide attempt

**S5: Sensitivity analysis: Negative-binomial regression model**

| Negative binomial regression model estimating effects of COVID-19 periods on suicide attempts including interaction effects | | | | |
| --- | --- | --- | --- | --- |
|  |  |  |  |  |
|  | RateRatio | 95% CI lower | 95%CI upper | p-value |
| **time-dependent** |  |  |  |  |
| *first-wave by Covid-19* | 3.130 | 1.361 | 7.198 | **.007** |
| *second-wave by Covid-19* | 1.506 | 0.685 | 3.313 | .309 |
| *Borderline personality disorder by Covid-19* | 3.369 | 1.226 | 9.256 | **.019** |
| *Substance use disorders by Covid-19* | 0.993 | 0.525 | 1.878 | .983 |
| *Schizophrenia and psychotic disorders by Covid-19* | 0.428 | 0.184 | 1.000 | .050 |
| *Bipolar and manic disorders by Covid-19* | 0.295 | 0.025 | 3.450 | .331 |
| *Depressive disorders by Covid-19* | 0.502 | 0.241 | 1.046 | .066 |
|  |  |  |  |  |
| **time-independent** |  |  |  |  |
| *Organic mental disorders* | 1.049 | 0.478 | 2.304 | .904 |
| *Substance use disorders* | 1.265 | 0.779 | 2.054 | .341 |
| *Schizophrenia and psychotic disorders* | 1.119 | 0.607 | 2.063 | .720 |
| *Bipolar and manic disorders* | 0.667 | 0.157 | 2.835 | .583 |
| *Depressive disorders* | 2740 | 1.625 | 4.619 | **<.001** |
| *Neurotic, somatoform and stress related disorders* | 0.978 | 0.655 | 1.459 | .913 |
| *Borderline personality disorder* | 1.190 | 0.492 | 2.875 | .699 |
| *Other personality disorders* | 1.846 | 1.014 | 3.359 | **.045** |
| *Age* | 1.001 | 0.990 | 1.012 | .882 |
| *gender* | 1.263 | 0.898 | 1.777 | .180 |

Results from the Negative-binomial regression model (sensitivity analysis) with the number of pED presentations after a suicide attempt per patient and observation period as outcome variable. Rate ratios greater than 1 indicate that a factor is increasing the number of presentations per patient after a suicide attempt. Rate ratios below 1 indicate that a factor is decreasing the number of presentations per patient after a suicide attempt. Abbreviations used: 95% CI = 95% confidence interval; Covid-19 = coronavirus disease 2019

**S6: Sensitivity analysis: Poisson regression model, excluding patients with multiple suicide attempts**

| Poisson model estimating effects of COVID-19 periods on suicide attempts including interaction effects; excluding patients with multiple SA | | | | |
| --- | --- | --- | --- | --- |
|  |  |  |  |  |
|  | RateRatio | 95% CI lower | 95%CI upper | p-value |
| **time-dependent** |  |  |  |  |
| *first-wave by Covid-19* | 3.232 | 1.424 | 7.336 | **.005** |
| *second-wave by Covid-19* | 1.509 | 0.694 | 3.280 | .299 |
| *Borderline personality disorder by Covid-19* | 2.961 | 1.091 | 8.034 | **.033** |
| *Substance use disorders by Covid-19* | 0.910 | 0.484 | 1.709 | .768 |
| *Schizophrenia and psychotic disorders by Covid-19* | 0.386 | 0.163 | 0.915 | **.031** |
| *Bipolar and manic disorders by Covid-19* | 0.308 | 0.027 | 3.535 | .345 |
| *Depressive disorders by Covid-19* | 0.477 | 0.231 | 0.983 | .045 |
|  |  |  |  |  |
| **time-independent** |  |  |  |  |
| *Organic mental disorders* | 1.008 | 0.465 | 2.185 | .984 |
| *Substance use disorders* | 1.264 | 0.786 | 2.032 | .334 |
| *Schizophrenia and psychotic disorders* | 1.105 | 0.606 | 2.013 | .745 |
| *Bipolar and manic disorders* | 0.652 | 0.156 | 2.728 | .558 |
| *Depressive disorders* | 2.698 | 1.622 | 4.488 | **<.001** |
| *Neurotic, somatoform and stress related disorders* | 0.928 | 0.623 | 1.382 | .712 |
| *Borderline personality disorder* | 1.179 | 0.498 | 2.794 | .708 |
| *Other personality disorders* | 1.876 | 1.054 | 3.339 | **.032** |
| *Age* | 1.002 | 0.991 | 1.013 | .737 |
| *gender* | 1.223 | 0.872 | 1.715 | .234 |

Results from the Poisson regression model (sensitivity analysis) with the number of pED presentations after a suicide attempt per patient and observation period as outcome variable; excluding patients with multiple pED presentations after a suicide attempt within one observation period. Rate ratios greater than 1 indicate that a factor is increasing the number of presentations per patient after a suicide attempt. Rate ratios below 1 indicate that a factor is decreasing the number of presentations per patient after a suicide attempt. Abbreviations used: 95% CI = 95% confidence interval; Covid-19 = coronavirus disease 2019

**S7: SA admissions requiring intensive care treatment**

|  | 2019 control period | First-wave | p-value | 2019/2020 control period | Second-wave | p-value |
| --- | --- | --- | --- | --- | --- | --- |
| **Total sample:** |  |  |  |  |  |  |
| *SA admissions with intensive care treatment (% of all SA admissions)* | 7 (36.8%) | 7 (19.4%) | 0.200 (Fisher) | 17 (22.1%) | 14 (17.5%) | 0.471 |
| *total SA admissions* | 19 | 36 |  | 77 | 66 |  |
| **BPD Subgroup:** |  |  |  |  |  |  |
| *SA admissions with intensive care treatment (% of all SA admissions)* | 2 (40.0%) | 2 (20.0%) | 0.560 (Fisher) | 1 (20.0%) | 4 (23.5%) | 1.000 (Fisher) |
| *total SA admissions* | 5 | 10 |  | 5 | 17 |  |

Comparison of SA admissions with intensive care treatment of the first-wave and the second-wave with their corresponding control periods in the total sample and in the BPD subgroup. P-values are resulting from chi²-tests and Fisher's-exact-test where indicated. Abbreviations used: BPD = Borderline personality disorder, SA = suicide attempt.

1. Kippe YD, Adam M, Finck A, Moran JK, Schouler-Ocak M, Bermpohl F, Gutwinski S, Goldschmidt T. Suicidality in psychiatric emergency department situations during the first and the second wave of COVID-19 pandemic. Eur Arch Psychiatry Clin Neurosci. 2022:1-13. doi: 10.1007/s00406-022-01486-6.
